# Understanding nursing perspective towards barriers to the optimal delivery of enteral nutrition in intensive care settings

**DOI:** 10.1186/s12912-024-01715-4

**Published:** 2024-01-15

**Authors:** Sara Zaher, Futoon AL. Sumairi, Sarah M. Ajabnoor

**Affiliations:** 1 Clinical Nutrition Department, Faculty of Applied Medical Sciences, Taibah University, P.O. Box 344, Madinah, 42353 Kingdom of Saudi Arabia; 2 Department of Clinical Nutrition, Faculty of Applied Medical Sciences, King Abdulaziz University, Jeddah, Saudi Arabia

**Keywords:** Enteral nutrition, EN barriers, PICU, ICU, Nurses

## Abstract

**Background:**

The management process of Enteral Nutrition (EN) typically involves the interaction between a team of health care practitioners. Nurses being the closest to the patients, have crucial responsibilities and play a major role in feeding delivery along with other medical treatments. This study was conducted to investigate the perception of the nurses working in adult and paediatric intensive care Units (ICUs) regarding the EN barriers and identify the factors that influenced their perception.

**Methods:**

The data in this cross-sectional study was collected via online survey between 15 October 2021 and January 2022. All nurses working in adult or paediatric ICUs across Saudi Arabia were eligible to participate. The tool used for the data collection was adapted from Cahill et al. (2016) and then reviewed and modified by the researchers. The survey collected information about the demographics of the nurses, and it included 24 potential EN barriers where the participants were asked to rate their importance on a scale from 1 to 5. Descriptive statistics were performed to describe the variables, univariant analysis were performed to compare the perceptions of the nurses regarding the EN barriers based on their characteristics followed by stepwise linear regression analysis.

**Results:**

A total of 136 nurses working in adult and paediatric ICUs were included in this study. The results showed that the most important barriers as perceived by the nurses was “*Frequent displacement of feeding tube, requiring reinsertion” [*3.29 ± 1.28], “*Delays in initiating motility agents in patients not tolerating enteral nutrition*” [3.27 ± 1.24] and “*Enteral formula not available on the unit”.* [3.27 ± 1.24]. Our results showed that the responses of the participants statistically varied based on their work settings, gender, region, and educational level for some items in the survey *(P-value* ≤ *0.05).* In the regression analysis, gender was the only variable statistically influenced the total Likert rating scores of the participants (*r* = -0.213, *p*-value = 0.013).

**Conclusion:**

This study identified several barriers that exist in the nursing practice of EN in critical care settings. There are distinct differences in the perception of the nurses to these barriers based on their characteristics. Understanding such differences is important for implementing future strategies for units that needed the most help in prioritizing EN delivery.

## Introduction 

Enteral nutrition (EN) is defined as the provision of nutrients via a tube directly to the gastrointestinal tract, to patients who are incapable of fulfilling their nutritional needs orally [1]. It is a standardised process that incorporates a multidisciplinary team (MDT) of physician, nurse, dietitian, and pharmacist [2–4]. This process involves comprehensive nutritional assessment, accurate prescription, proper administration, and frequent monitoring and re-evaluation according to the patient’s condition [5, 6].

The management process of EN typically involves the interaction between a team of health care practitioners consisting of physician, nurse specialist, dietitian, and pharmacist [7]. In this MDT, each member has varying responsibilities according to their specialities and practice. Effective communication and collaborative efforts among the MDT are crucial for achieving optimum health outcomes [3]. The physician’s role relies on the overall understanding of the patient’s medical condition, diagnostics, prognostics, and medical treatment, as well as coordinating the medical team [8, 9]. Dietitians play a central part in the provision of EN to patients, as their role starts from the development and implementation of EN hospital protocols [8, 9]. In addition, the dietitians are responsible for the assessment of patient’s nutritional status and needs as well as recommending and overseeing the EN feeding plan that attains to patients’ needs patients’ needs [8, 9].

Nurses being the closest to the patients, have crucial responsibilities and play a major role in feeding delivery along with other medical treatments [8, 10]. Their responsibilities begin with nutritionally screening patients, inserting and assessing the placement of the feeding tube, ensuring proper handling of the feeding formula, and performing hygiene and care procedures such as water flushing and sputum suction [11]. During the process of oral care with sputum suction the EN should be withheld to prevent choking. The nurses are the key persons responsible for re-starting the feed promptly after finishing this process to avoid the risk of underfeeding [11]. Furthermore, nurses are also responsible for implementing the EN plan through feeding the patient, assuring adequate nutritional delivery, and frequently monitoring the patient’s tolerance [10]. Al-Sayaghi et. al. has indicated that critical care nurses demonstrated a low level of knowledge and responsibility regarding EN [12]. This highlights the urgent need of studies to understand the nurse’s perception about EN and the barriers to achieve adequate feeding. In addition, nurses must be engaged to be part of the multidisciplinary nutritional support team with clear roles and responsibilities.

In previously published work it has been reported that around 60% of the prescribed caloric intake for patients in the ICU is not being delivered via the enteral route due to avoidable barriers that consequently result in either failure or delay in achieving optimal nutritional goal [13–16]. Since nurses are continuously involved with patients' care plan it is crucial to understand and investigate their perception regarding the barrier to EN delivery in the ICU [13–16]. Since nurses are continuously involved with patients' care plan it is crucial to understand and investigate their perception regarding the barrier to EN delivery in the ICU. Thus, this study was conducted to investigate the perception of the nurses working in adult and paediatric intensive care settings regarding the EN barriers, compare the perception of the nurses working in adults and those working in paediatric ICUs regarding EN barriers and finally, we also aim to identify the factors that influenced their perception regarding these EN barriers.

## Methods

### Study design, participants and data collection

The design of the current study was cross-sectional, where all nurses currently practicing in adult or paediatric ICUs working in both working in governmental or private hospitals in Saudi Arabia were invited to participate in the study. All other healthcare professionals were excluded. The data was collected through online survey between 15 October 2021 and January 2022. The survey was initially promoted on various social media platforms (e.g., WhatsApp and Twitter). Then, a Chain-referral sampling was performed where nurses known to the investigators from all regions of the kingdom were contacted to achieve adequate convenience sample of nurses working in intensive care settings.

### Assessment of EN barriers as perceived by the nurses

The tool used to assess the perception of the nurses regarding the EN barriers in intensive care settings was adapted from Cahill et al. (2016) [17]. Two experts in the field of nutrition support modified and validated the tool. Detailed description of the tool used in this study can be found in Zaher et al. (2022) [18]. In brief, two questions concerning the demographic characteristics of the participants were added, and some questions were rewritten to improve clarity. The survey was finally reviewed and adjusted based on the feedback received from the nurses involved in the pilot testing of the survey. The data collected from the nurses who participated in the pilot testing was excluded from the current analysis.

The survey consisted of two parts, the first part collected information about the demographics of the participants and the second part included 24 potential EN barriers where the nurses were asked to rate the items’ importance as EN barrier on a scale from 1 (not at all important), 2 (slightly important), 3 (important), 4 (Fairly important) to 5 (very important).

The survey included Five domains to categories the EN barriers: The first domain included two questions about the guidelines and recommendations, the second domain included seven questions about EN delivery to patients, the third domain included three questions about the intensive care resources, the fourth domain included seven questions about the attitudes and behaviours of critical care providers, and finally, the fifth domain included five questions about dietitian’s resources in ICUs. The result of the Cronbach's alpha test indicated a good internal reliability of the tool (0.944).

### Statistical analysis

Data was analysed using the “Statistical Package for Social Sciences” software version 28 (SPSS Inc.) (SPSS 28, SPSS Inc., Chicago, IL, USA). To assess the data normality of the continuous variables we used Shapiro–Wilk test. Data was presented as Frequencies and percentages to describe the data. Continues variables were presented as mean ± standard deviation (SD). Mean (± SD) was calculated to determine the most and least important EN barriers as perceived by the nurses included in the study. A total Likert rating score of the 24 EN barriers included in the survey was calculated for each participant to be used in further statistical tests.

Mann–Whitney U test and Kruskal–Wallis test were performed to compare the perceptions of the nurses regarding the EN barriers based on the participants characteristics such as their work, settings, sex, region, and educational levels.

A stepwise linear regression analysis was performed to identify factors that influenced the perceptions of the nurses who participated in the study regarding the EN barriers encountered in intensive care settings. The outcome variable in the regression model was the total Likert rating score of the 24 EN barriers. Multiple independent variables were added to the models including the gender, educational level, work setting, years of experience, type of health care facility and region.

## Results

A total of 136 nurses working in adult and paediatric ICUs across Saudi Arabia participated in this study. Most of the participants were females (*n* = 103, 75%). Most of the responses were received from the Western region, and their mean years of work experience in intensive care settings was 4.1 ± 3.06 years. The characteristics of the study participants are presented in Table 1.

**Table 1:** General characteristics of the study participants

		**Adults ICU nurses**	**PICU nurses**	**All Participants**
		**(*n* = 83)**	**(*n* = 53)**	**(*n* = 136)**
**Gender**	*Female*	55 (66.26%)	48 (90.56%)	103 (75.73%)
	*Male*	28 (33.73%)	5 (9.43%)	33 (24.26)
**Region**	*Western region*	44 (53%)	34 (64.21%)	78 (57.35%)
	*Eastern region*	8 (9.6%)	6 (11.32%)	14 (10.29%)
	*Central region*	23 (27.7%)	8 (15.10%)	31 (22.79%)
	*Southern region*	4 (4.8%)	4 (7.5%)	8 (5.88%)
	*Northern region*	4 (4.8%)	1 (1.9%)	5 (3.68%)
**Education and training**	*Diploma*	1 (1.2%)	2 (3.8%)	3 (2.21%)
	*Intern*	18 (1.2%)	2 (3.8%)	20 (14.71%)
	*Bachelor’s*	52 (62%)	48 (90.6%)	100 (73.53%)
	*Master’s*	7 (8.4%)	0 (0%)	7 (5.15%)
	*Doctorate*	2 (2.4%)	0 (0%)	2 (1.47%)
	*Other (Residency, Fellowship, Board)*	3 (3.6%)	1 (0%)	4 (2.94%)
**Type of health care facility**	*University teaching hospitals*	2 (2.4%)	1 (1.9%)	3 (2.21%)
	*Specialized hospitals*	4 (4.8%)	1 (1.9%)	5 (3.68%)
	*Private hospitals*	9 (10.8%)	2 (3.8%)	11 (8.09%)
	*National guard hospitals*	1 (1.2%)	0	1 (0.74%)
	*Ministry Of Health (MOH) hospitals*	57 (68.7%)	43 (81.1%)	100 (73.53%)
	*Military hospitals*	7 (8.4%)	3 (5.7%)	10 (7.35%)
	*Medical cities*	3 (3.6%)	3 (5.7%)	6 (4.41%)
**Years of experience**	**Mean (± SD)**	5.7 (± 3.6)	6.5 (± 3.6)	5.8 (± 3.95)
	**Median (IQR)**	2 (6–1)	5 (7.7–3)	5 (1–10)
	*1- 5 years (n)*	50 (60%)	22 (41.5%)	72 (52.94%)
	*5- 10 years (n)*	19 (23%)	14 (26%)	33 (24.26%)
	*10* + *(n)*	14 (17%)	17 (32%)	31 (22.79%)

*• Data presented as frequencies and percentage*

We calculated the mean (± SD) and the median (IQR) to determine the most and least important barriers to EN as perceived by the nurses working in adult and paediatric ICUs. The results showed that the most important barrier was “*Frequent displacement of feeding tube, requiring reinsertion” [*3.29 ± 1.28, 3 (2–5)] followed by “*Delays in initiating motility agents in patients not tolerating enteral nutrition*” [3.27 ± 1.24, 3 (2–4.75)] which were both included in the “delivery of EN” domain. The third most important barrier was “*Enteral formula not available on the unit”.* [3.27 ± 1.24, 3 (2–4.75)] which was included in the “ICU/PICU resources” domain. On the other hand, the least important barriers as reported by the nurses were “*non-ICU physicians (i.e., surgeons, gastroenterologists) requesting patients not be fed enterally*” [2.94 ± 1.24, 3 (2–4)] preceded by “*Nurses failing to progress feeds as per the feeding protocol”* [3.00 ± 1.25, 3 (2–4)]; both barriers were included in the “critical care providers attitude and behaviour” domain. A Kruskal–Wallis test was then performed to compare the difference in Likert ratings score of the 5 domains, and no statistical difference was recorded between the score of the 5 domains (*P*-value = 0.284) (Table 2).

**Table 2:** Description of enteral feeding barriers as perceived by the nurses working in adults and paediatric ICUs

Questions	**All participants Likert rating score Mean (± SD)**	**Adult ICU nurses Likert rating score Mean (± SD)**	**PICU nurses Likert rating score Mean (± SD)**	**Comparison between nurses working in adults and paediatric ICUs. (*P*-value)**
Domain 1				
** Current scientific evidence supporting some nutrition interventions is inadequate to inform practice**	3.25 ± 1.19	3.1 ± 1.066	3.49 ± 1.265	0.053
*** Lack of feeding protocol in place to guide the initiation and progression of enteral nutrition in your institution***	3.19 ± 1.25	3.05 ± 1.188	3.42 ± 1.322	0.095
**Mean ± SD Likert rating for Domain 1**	3.24 ± 1.23			
**Domain 2**				
*** Delay in physician ordering initiation of enteral nutrition***	3.09 ± 1.19	2.98 ± 1.158	3.26 ± 1.195	0.164
*** Waiting for physician/radiology to read x-ray and confirm tube placement***	3.10 ± 1.27	3.11 ± 1.21	3.09 ± 1.39	0.95
*** Frequent displacement of feeding tube, requiring reinsertion***	3.29 ± 1.28	3.28 ± 1.233	3.32 ± 1.37	0.847
*** Delays in initiating motility agents in patients not tolerating enteral nutrition (ie, high gastric residual volumes)***	3.27 ± 1.24	3.24 ± 1.226	3.32 ± 1.283	0.717
*** Delays and difficulties in obtaining small bowel access in patients not tolerating enteral nutrition (i.e., high gastric residual volumes)***	3.22 ± 1.23	3.22 ± 1.25	3.23 ± 1.235	0.965
*** In resuscitated, hemodynamically stable patients, other aspects of patient care still take priority over nutrition***	3.13 ± 1.51	3.04 ± 1.12	3.26 ± 1.211	0.264
*** Nutrition therapy not routinely discussed on patient care rounds***	3.10 ± 1.18	2.93 ± 1.135	3.36 ± 1.226	0.038*
**Mean ± SD Likert rating for Domain 2**	3.22 ± 1.25			
**Domain 3**				
*** Not enough nursing staff to deliver adequate nutrition***	3.33 ± 1.27	3.25 ± 1.208	3.45 ± 1.367	0.373
*** Enteral formula not available on the unit***	3.27 ± 1.26	3.17 ± 1.257	3.43 ± 1.264	0.233
*** No or not enough feeding pumps on the unit***	3.30 ± 1.3	3.25 ± 1.351	3.38 ± 1.244	0.59
**Mean ± SD Likert rating for Domain 3**	3.18 ± 1.32			
**Domain 4**				
*** Non-ICU physicians (i.e., surgeons, gastroenterologists) requesting patients not be fed enterally***	2.94 ± 1.21	3.02 ± 1.22	2.81 ± 1.194	0.561
*** Nurses failing to progress feeds as per the feeding protocol***	3.00 ± 1.25	3.2 ± 1.266	3.08 ± 1.253	0.141
*** Feeds being held due to diarrhea***	3.03 ± 1.19	3.08 ± 1.128	2.94 ± 1.307	0.506
*** Fear of adverse events due to aggressively feeding patients***	3.18 ± 1.25	3.23 ± 1.253	3.09 ± 1.26	0.543
*** Feeding being held too far in advance of procedures or operating room visits***	3.24 ± 1.20	3.29 ± 1.195	3.17 ± 1.221	0.574
*** General belief among ICU team that provision of adequate nutrition does not impact on patient outcome***	3.13 ± 1.29	3.11 ± 1.288	3.15 ± 1.321	0.853
*** Lack of familiarity with current guidelines for nutrition in the ICU***	3.26 ± 1.26	3.23 ± 1.337	3.3 ± 1.153	0.744
**Mean ± SD Likert rating for Domain 4**	3.09 ± 1.28			
**Domain 5**				
*** Waiting for the dietitian to assess the patient***	3.22 ± 1.17	3.19 ± 1.184	3.26 ± 1.163	0.73
*** Dietitian not routinely present on weekday patient rounds***	3.21 ± 1.22	3.3 ± 1.207	3.08 ± 1.253	0.297
*** Not enough dietitian time dedicated to the ICU during regular weekday hours***	3.15 ± 1.14	3.25 ± 1.157	3.00 ± 1.109	0.208
*** No or not enough dietitian coverage during evenings, weekends, and holidays***	3.21 ± 1.27	3.22 ± 1.288	3.21 ± 1.261	0.967
*** There is not enough time dedicated to education and training on how to optimally feed patients***	3.17 ± 1.15	3.16 ± 1.184	3.19 ± 1.128	0.876
**Mean ± SD Likert rating for Domain 5**	3.16 ± 1.25			
**Comparison between the 5 domains (*P* value)**	0.284			

*• Data presented as frequencies and percentage*

*• Mann–Whitney U test was conducted to compare the median the Likert rating score of each barrier between nurses working in adult and pediatric ICU*

*• Kruskal Wallis test was conducted to compare the median of the 5 domains*

^*^P value is statistically significant at < 0.05 level

A series of Mann–Whitney U tests were performed to compare the perceptions of the nurses who participated in the study regarding the importance of each item as a barrier to EN based on their work setting (adult or paediatric ICU). No significant differences were recorded between the responses of the nurses working in adult ICUs and those working in PICUs except for one item “*Nutrition therapy not routinely discussed on patient care rounds”*, *P*-value = 0.038, (Table 2).

We then compared the responses of the study participants regarding the importance of each item as barrier to EN based on their gender. Our results showed that the responses of the participants statistically varied based on their gender for the following items; “*Lack of feeding protocol in place to guide the initiation and progression of enteral nutrition in your institution”,* (*P*-value = 0.029*), “Delay in physician ordering initiation of enteral nutrition”,* (*P*-value = 0.022*), “Delays in initiating motility agents in patients not tolerating enteral nutrition”,* (*P*-value = 0.044), *“In resuscitated, hemodynamically stable patients, other aspects of patient care still take priority over nutrition”,* (P-value = 0.016*), “Nutrition therapy not routinely discussed on patient care rounds”,* (*P*-value = 0.033), *“Non-ICU physicians (i.e., surgeons, gastroenterologists) requesting patients not be fed enterally”,* (*P*-value = 0.008) *and “Waiting for the dietitian to assess the patient”,* (*P*-value = 0.003), (Table 3).

**Table 3:** Comparison between the perception of male and female nurses working in adults and paediatric ICUs regarding EN barriers

Questions	**Female nurses Likert rating score Mean (± SD)**	**Male nurses Likert rating score Mean (± SD)**	***P*-value**
**Domain 1**	***n* = 103**	***n* = 33**	
**Current scientific evidence supporting some nutrition interventions is inadequate to inform practice**	3.34 ± 1.099	2.97 ± 1.311	0.095
**Lack of feeding protocol in place to guide the initiation and progression of enteral nutrition in your institution**	3.32 ± 1.238	2.79 ± 1.219	0.029*
**Domain 2**			
*** Delay in physician ordering initiation of enteral nutrition***	3.21 ± 1.185	2.70 ± 1.075	0.022*
*** Waiting for physician/radiology to read x-ray and confirm tube placement***	3.22 ± 1.313	2.73 ± 1.098	0.046
*** Frequent displacement of feeding tube, requiring reinsertion***	3.42 ± 1.295	2.91 ± 1.182	0.053
*** Delays in initiating motility agents in patients not tolerating enteral nutrition (ie, high gastric residual volumes)***	3.4 ± 1.183	2.88 ± 1.364	0.044*
*** Delays and difficulties in obtaining small bowel access in patients not tolerating enteral nutrition (i.e., high gastric residual volumes)***	3.31 ± 1.221	2.94 ± 1.273	0.145
*** In resuscitated, hemodynamically stable patients, other aspects of patient care still take priority over nutrition***	3.25 ± 1.161	2.73 ± 1.069	0.016*
*** Nutrition therapy not routinely discussed on patient care rounds***	3.21 ± 1.234	2.73 ± 0.944	0.033*
**Domain 3**			
*** Not enough nursing staff to deliver adequate nutrition***	3.45 ± 1.274	2.97 ± 1.212	0.049
*** Enteral formula not available on the unit***	3.31 ± 1.321	3.15 ± 1.064	0.404
*** No or not enough feeding pumps on the unit***	3.4 ± 1.316	3.00 1.25	0.097
*** Non-ICU physicians (i.e., surgeons, gastroenterologists) requesting patients not be fed enterally***	3.1 ± 1.249	2.45 ± 0.938	0.008*
*** Nurses failing to progress feeds as per the feeding protocol***	3.24 ± 1.248	2.88 ± 1.269	0.141
*** Feeds being held due to diarrhea***	3.09 ± 1.214	2.85 ± 1.149	0.247
*** Fear of adverse events due to aggressively feeding patients***	3.24 ± 1.248	2.97 ± 1.262	0.250
*** Feeding being held too far in advance of procedures or operating room visits***	3.3 ± 1.179	3.06 ± 1.273	0.234
*** General belief among ICU team that provision of adequate nutrition does not impact on patient outcome***	3.22 ± 1.328	2.82 ± 1.158	0.099
*** Lack of familiarity with current guidelines for nutrition in the ICU***	3.33 ± 1.271	3.03 ± 1.237	0.153
**Domain 4**			
*** Waiting for the dietitian to assess the patient***	3.38 ± 1.156	2.73 ± 1.098	0.003*
*** Dietitian not routinely present on weekday patient rounds***	3.26 ± 1.236	3.06 ± 1.197	0.344
*** Not enough dietitian time dedicated to the ICU during regular weekday hours***	3.18 ± 1.169	3.06 ± 1.059	0.465
*** No or not enough dietitian coverage during evenings, weekends, and holidays***	3.25 ± 1.266	3.09 ± 1.308	0.470
*** There is not enough time dedicated to education and training on how to optimally feed patients***	3.24 ± 1.167	2.94 ± 1.116	0.130

*• Mann–Whitney U test was conducted to compare the median the Likert rating score of each barrier between male and female nurses working in adult and pediatric ICU*

^*^P value is statistically significant at < *0.05 level*

Kruskal Wallis test was performed to compare the perceptions of the nurses regarding the importance of each item as a barrier to EN according to the region that they are based in. The results showed that the responses of the participants statistically varied between regions for the following items; “*Not enough nursing staff to deliver adequate nutrition”,* (*P*-value = 0.034), *“Enteral formula not available on the unit”,* (*P*-value = 0.037) *both items included in ICU/PICU resources.* A statistical difference was also recorded between the responses of the participants for the item “*General belief among ICU team that provision of adequate nutrition does not impact on patient outcome”*, (*P*-value = 0.044)*.* The results also showed that the responses of the participants statistically varied based on their educational level for the following items; “*Current scientific evidence supporting some nutrition interventions is inadequate to inform practice”, (P-value* = *0.01), “Delay in physician ordering initiation of enteral nutrition”,* (*P*-value = 0.025), *“Delays in initiating motility agents in patients not tolerating enteral nutrition”,* (*P*-value = 0.027), “*Dietitian not routinely present on weekday patient rounds”,* (*P*-value = 0.031), “*There is not enough time dedicated to education and training on how to optimally feed patients”,* (*P*-value = 0.021).

The total Likert rating scores of the 24 items were calculated for each participant. The results showed that participants had a mean ± SD Likert rating score of 76.44 ± 20.10. A stepwise linear regression analysis was performed to identify factors influencing the nurses’ perceptions regarding EN barriers in intensive care settings. In the regression model the total Likert rating scores of the 24 items was used as the outcome variable, while the independent variables in the model were the characteristics of the participants including gender, work settings, years of experience, educational level, the region of the kingdom where they are practicing and the type of health care facility they worked in. The regression analysis indicated that gender was the only variable statistically influenced the total Likert rating scores of the participants (*r* = -0.213, *p*-value = 0.013). The female participants appeared to have higher Likert rating scores compared to male participants (Table 4). In the sub-analysis of the cohort working in adults intensive care units, the region statistically influenced the total Likert rating scores of the participants (*r* = -0.275, *p*-value = 0.012), (Table 4).

**Table 4:** Regression analysis to identify the factors influenced the perception of the nurses working in adults and paediatrics ICUs regarding the EN barriers

Combined sample (Nurses working in Adult ICUs and PICUs)			
**Model 1 Outcome variable:** Total score	**R**	**R**^**2**^	**Adjusted R**^**2**^
	0.213	0.046	0.038
***Dependent variable (n* = *136)***	***Beta***		***P-value***
Gender ^a^	-0.213		0.013*
Work settings (adult ICU or PICU) ^b^	-0.019		0.829
Years of experience ^b^	-0.02		0.816
Educational level ^b^	-0.132		0.128
Region ^b^	0.14		0.108
Type of health care facility ^b^	0.059		0.496
**Nurses working in Adult ICUs**			
**Model 2 Outcome variable:** Total score	**R**	**R**^**2**^	**Adjusted R**^**2**^
	0.275	0.076	0.064
***Dependent variable (n* = *83)***	***Beta***		***P-value***
Region ^a^	0.275		0.012*
Gender ^b^	-0.133		0.236
Years of experience^b^	-0.101		0.369
Educational level ^b^	-0.171		0.126
Type of health care facility ^a^	0.209		0.061
**Nurses working in PICUs**			
**Model 3 Outcome variable:** Total score	**R**	**R**^**2**^	**Adjusted R**^**2**^
	0.383	0.147	0.130
***Dependent variable (n* = *53)***	***Beta***		***P-value***
Gender ^a^	-0.383		0.005*
Years of experience^b^	0.14		0.323
Educational level ^b^	0.015		0.919
Region ^b^	0.001		0.996
Type of health care facility ^b^	-0.089		0.531

Coding

• Gender (female coded as 1 and male coded as 2)

• Work setting (adult ICU coded as 1 and PICU coded as 2)

• Educational level (diploma coded as 1, intern coded as 2, bachelor’s coded as 3, master’s coded as 4, residency coded as 5, fellowship coded as 6, and doctorate coded as 7)

• Type of health care facility (medical city coded as 1, military hospital coded as 2, ministry of health hospital coded as 3, national guard hospital coded as 4, private hospital coded as 5, specialised hospital coded as 6, and university teaching hospital coded as 7)

• Region (Central region coded as 1, Eastern region coded as 2, Northern region coded as 3, Southern region coded as 4, and Western region coded as 5)

^a^Predictors: (constant)

^b^Excluded variables

^*^P value is statistically significant at < *0.05 level*

## Discussion

The present study aimed to investigate nurses’ perception toward EN barriers in adult and paediatric intensive care settings in Saudi Arabia and to explore the factors influencing their perception. Most of the included nurses in this study were females. The most important reported barriers were the ones associated with EN delivery and with resources’ availability in critical care settings. While the least important barriers were the ones related to the critical care provider attitudes and behaviours. However, the absence of routine discussion of nutritional therapy during ward rounds was the only barrier that was significantly different between nurses working in adult ICUs and those working in PICUs. Moreover, the results of the univariate analysis showed the nurses' responses to some barriers statistically varied according to sociodemographic characteristics. Overall, findings from the multi-linear regression analysis showed that gender was the only variable that statistically influenced the overall rating scores of the nurses’ perception of EN barriers. Female nurses appeared to have higher rating scores of perceived EN barriers than males. The geographical region of the workplace also influenced the total rating scores of perceived barriers particularly for nurses practicing in adult ICU.

Identifying barriers related to EN delivery is essential to optimize nursing practice in critical care settings, which will help in achieving patients’ nutrient requirements and caloric targets. In this study, one of the main barriers indicated by the nurses is the issue of frequent tube displacement and reinsertion, which could lead to prolonged periods of feeding interruptions. According to recent studies, the most frequently reported causes of EN interruptions in patients admitted to ICU settings are diagnostic tests (i.e., radiological procedures and gastric residual volume (GRV) evaluation) and problems with feeding tubes [19, 20]. The increased number of EN disruption episodes was shown to be associated with a higher mortality rate [20]. The issue of delaying the initiation of motility medications in patients not tolerating EN was also identified as a main barrier in this study. This barrier was ranked as one of the top ten EN barriers by an earlier investigation of ICU nurses working in North American countries [21]. Gastrointestinal dysmotility is common among patients in the ICU [22], which can make EN feeding difficult to deliver. However, earlier administration of motility agents is recommended for effective EN therapy in the ICU [23].

Another identified barrier in the current study that can have a significant impact on patient care is the unavailability of appropriate EN formulations in the unit. Findings from similar studies reported that resource availability in terms of formula availability is a commonly perceived barrier to EN practice by nurses [24]. Such barriers can contribute to suboptimal delivery of EN and hinder patient recovery. Overall, critically ill patients in ICUs are at a significant risk of acquiring malnutrition, which is linked to a worsened clinical prognosis [25].

Regarding the critical care provider attitudes and behaviours toward EN practice, two barriers related to this domain were reported in this study as least important. Institutional-related factors can play a role in enhancing EN practices in the ICU. A supportive ICU workplace that values and prioritizes nutritional care can positively influence nurses' attitudes and behaviours toward EN [26]. This may include having established protocols and guidelines, promoting nursing education regarding EN's effect on patient outcomes, and supporting interdisciplinary collaboration to facilitate consistent and evidence based EN practices [26]. While inconsistent practices such as non-ICU physicians requesting patients not to be fed via EN can hinder nurses' ability to provide optimal EN care.

On the other hand, there is a degree of variation in nurses’ perspectives toward some EN barriers between adult ICU and PICU nurses. In this study, the absence of routine discussion of nutritional therapy during ward rounds was the only barrier that was significantly different between nurses working in adult ICUs and those working in PICUs. In general, the patient population in the PICU is considered heterogeneous, therefore, it is recommended to implement individualized nutrition support that is based on the patient's baseline characteristics and requirements [27]. Indeed, the level of interprofessional collaboration and communication regarding EN practices could differ between both settings. Based on findings from an international nursing survey, only a few PICUs have an established multidisciplinary nutritional support team [28]. The availability of a nutritional support team may benefit nurses’ education in nutrition and help facilitate comprehensive nutritional guidance and decision-making [28].

Nurses’ perceived barriers to EN practice in critical care setting is considered multifactorial and could vary across different hospitals, thus, understanding the factors associated with these barriers is highly important. In this study, gender was found to influence nurses' perceptions of EN barriers. Traditionally, nursing has been a female-dominated profession, which explains why most of the participants who completed the survey were females. However, the small number of male nurses (*n* = 32) included in the study is still considered statistically acceptable to measure the impact of gender differences on the perception score of EN barrier. Previous studies have shown no difference between both genders regarding the overall score of perceived barriers [29, 30]. On the contrary, the present study reported that female nurses perceived more EN barriers than male nurses. While both male and female nurses might acquire the same level of knowledge and skills, societal expectations may result in male nurses being perceived as more confident in managing practice-related barriers and consequently perceiving fewer barriers [31]. Nevertheless, the awareness of female nurses with the perceived EN barriers observed in the present study might be developed through their clinical experience, frequent application of evidence-based practice, or continuous involvement in lifelong learning. According to Silberman et al., the provision of EN continuous education program led to a considerable improvement in the knowledge of EN practice among ICU nurses [32]. It might also be attributed to the fact that the nutrition practice is usually female dominant and therefore female nurses might have more interest and awareness in the nutrition-related practice [33].

Another demographic factor influencing nurses’ perception of EN barriers was the geographical region of nurses’ workplaces. Nurses working in smaller regions of Saudi Arabia (i.e., Southern and Northern regions) perceived more EN barriers that relate to the availability of staff for EN delivery and the availability of EN formula. This is consistent with the national trend of the nursing shortage, which is considered one of the challenges that Saudi Arabia is experiencing [34]. Although specific regional challenges might contribute to the variation in perceived EN barriers, findings from regional studies are missing. The nursing practice in Saudi Arabia has usually been facing several challenges including the shortage issue of nurses in smaller regions [35]. Additionally, the limited availability of well-established tertiary hospitals with enough medical resources like enteral formulas in smaller regions of Saudi Arabia could result in more challenges faced by nurses working in critical care settings. Darawad et al. found that nurses in large educational hospitals indicated fewer barriers than nurses from private hospitals [29]. Thus, institutional-related factors could influence nurse’s perception of EN barriers. Currently, the Saudi Vision 2030 program is having an impact on advancing the nursing profession across all regions via the ongoing changes and transformation in the country’s healthcare system [36].

To our knowledge, this study is considered the first one to investigate nurses’ perceptions regarding EN barriers in critical care settings in Saudi Arabia. Also, it is considered the first study to explore the difference between the perception of the nurses working in adult ICUs and those working in PICUs. Because nursing in Saudi Arabia is considered a developing healthcare profession with a high shortage rate of local nurses [37], previous reports concerning nursing practice in critical care settings were focused on investigating other more apparent barriers than the one concerning EN, which involved nurses’ perception regarding pain management [38], pressure injury prevention [39], shift handover and communication practice [40], and patient advocacy [41]. However, the most reported EN barriers in this study (i.e., barriers related to EN delivery and availability of formula) were relatively aligned with the international trend of nurses’ perceptions of EN barriers in ICU [42, 43].

Nevertheless, a few limitations were found to be associated with the present study. Even though the study included nurses from several healthcare sectors in Saudi Arabia, the small sample size limits the generalizability of its findings. Future research should use a bigger sample size. Also, the high percentage of females and nurses residing in the western region who responded to the survey, might further limit the generalizability of the results. Therefore, study findings may not accurately represent the diverse perspectives of nurses working in critical care settings in Saudi Arabia. Additionally, the use convenient sampling via social media platforms could be a potential selection bias, however, according to the national statistics it is estimated that over than 80% of the Saudi population have internet access and use social media [44, 45]. Another limitation is related to the study design, which only was able to assess nurses' perceptions of EN barriers without evaluation of associated patient outcomes. Future studies in this area should try to include patient outcomes (e.g., length of ICU stay, EN complications, and whether target nutritional requirements are met) and correlate it with nurses’ perceived EN barriers. This will help to better understand the key EN barriers that need improvement by the nursing practice in critical care settings.

## Conclusion

In conclusion, numerous barriers exist in the nursing practice of EN in adult and paediatric critical care settings. Such barriers can impede the effective implementation and delivery of EN, compromising patient outcomes. It is crucial for the healthcare workforce in Saudi Arabia to address these barriers by providing ongoing education and training to nurses, improving staffing levels for local nurses across all regions, improving gender distribution, and ensuring a supportive environment in hospitals (e.g., supporting interdisciplinary collaboration) for optimal nutritional care. This will enable nurses to overcome these barriers and deliver optimal EN to critically ill patients. While EN is a crucial aspect of nursing care in both adult ICU and PICU settings, there are distinct differences in the barriers encountered by nurses. Understanding such differences is important for implementing future strategies for units that needed the most help in prioritizing EN delivery. Moreover, sociodemographic factors could influence the nursing practice of EN. By recognizing and addressing these factors, healthcare organizations across Saudi Arabia can create an environment that facilitates the effective implementation of EN protocol in the ICU.

## Data Availability

The datasets used and/or analysed during the current study are available from the corresponding author on reasonable request.
